# Fabrication and Composition of MA_1–*y*_FA_*y*_SnI_3–*x*_Br_*x*_ Thin Films for Lead-Free Perovskite Solar Cells

**DOI:** 10.1021/acsomega.4c00535

**Published:** 2024-08-05

**Authors:** Yuto Sasaki, Mariko Murayama, Xinwei Zhao

**Affiliations:** Department of Physics, Graduate Faculty of Science, Tokyo University of Science, Tokyo 162-8601, Japan

## Abstract

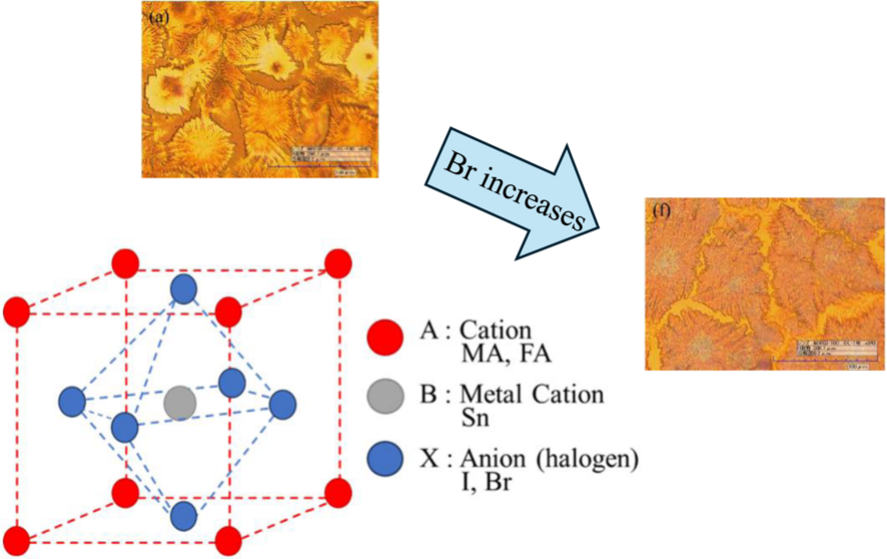

Perovskite solar cells have gained significant attention in recent years due to their lightweight nature, flexibility, and ability to generate power even in weak-light conditions. Despite these advantages, the current mainstream perovskite solar cells contain lead, raising concerns about their environmental and human health effects. Tin is expected to be a substitutional element for lead; however, tin-based perovskite solar cells currently have low power conversion efficiency. Altering the composition of the perovskite is crucial for enhancing its performance. In this study, perovskite solar cells with mixed MA/FA and I/Br components were designed and fabricated based on the calculation of the tolerance factor. The crystallinity and band gap of perovskite thin films were manipulated by changing the compositions of anions and cations. A suitable composition ratio for perovskite solar cells was proposed and discussed.

## 1 Introduction

Solar cells are one of the most important renewable energy sources to address energy scarcity and global environmental issues.^1,2^ Perovskite solar cells have achieved a power conversion efficiency (PCE) comparable to that of silicon-based solar cells and are being actively researched. In recent years, perovskite solar cells have attracted great attention, and their PCE has leaped from 3.8% in 2009 to above 25% nowadays.^3−6^

Currently, lead (Pb)-based perovskite solar cells are the mainstream, and there are concerns about Pb contamination of the environment and the human health.^1^ Therefore, it is necessary to develop Pb-free perovskite solar cells, and tin (Sn) is expected to replace Pb.^7,8^ However, Sn-based perovskite solar cells have a lower PCE than Pb-based ones. Thus, there is a need to improve the PCE of Sn-based perovskite solar cells. It is important to improve the crystallinity and optimize the band gap of perovskite crystals to absorb the sunlight with a wider range of wavelengths, including visible region. The optimal band gap value for single-junction solar cells is estimated to be 1.34 eV.^2,7^ Sunlight consists of 6% ultraviolet (UV) light, 50% visible light, and 44% infrared light. By narrowing the band gap, solar cells can absorb sunlight up to the near-infrared (IR) region, which enables the conversion of more light energy into electrical energy. However, too small a band gap gives the limitation of the extractable voltage; hence, an optimal band gap with a good balance between absorbing light over the range of UV-IR wavelength and the extractable voltage is important.

The perovskite materials can be written as ABX_3_, where A is CH_3_NH_3_^+^ (MA^+^) or CH(NH_2_)_2_^+^ (FA^+^), B is the metal cation (Pb^2+^ or Sn^2+^), and X is the halide anion (I^–^, Br^–^, or Cl^–^).^9,10^ These halogen perovskite materials are highly ion-conductive crystals containing highly electronegative halogen ions, which can diffuse through the solid with high mobility.^7,11^

In perovskite crystals, the ionic radii of compositing atoms will affect the stability of the crystal lattice structure, which is indicated by the tolerance factor *t* as
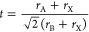
where *r*_A_ and *r*_B_ are the ionic radii of A and B site cations, and *r*_X_ is the ionic radius of anion X. For *t* in the range of 0.9 ≤ *t* < 1, the crystal exhibits a perovskite structure.^12^ Specifically, in the range of 0.95 ≤ *t* < 1, the crystal is expected to show an ideal cubic structure.^11^ Whereas for *t* in the range of 0.71 ≤ *t* < 0.9, the orthorhombic, rhombohedral, or tetragonal structure could be formed. Furthermore, for *t* in the range of *t* < 0.71 and *t* > 1, it rarely shows perovskite structure because its structure is too distorted.^12^

Halogen perovskites show significantly different optical absorption wavelengths depending on the selected halogen ions, and their band gaps can be tuned by adjusting the atomic composition. In mixtures of iodine (I) and bromine (Br), the band gap has been found to depend linearly on the mixing I/Br ratio.^13^

In this study, we first calculated the crystallinity and band gap of MA_1–*y*_FA_*y*_SnI_3–*x*_Br_*x*_, with the aim of determining the optimal composition ratio to enhance the PCE of Sn-based perovskite solar cells. Subsequently, we prepared samples near the optimal ratio and assessed their crystallinity and band gap, and then the best composition ratio for device application was proposed and discussed.

## 2 Experiments

### 2.1 Film Formation

MA_1–*y*_FA_*y*_SnI_3–*x*_Br_*x*_ thin films with compositions suitable for perovskite crystals were fabricated based on the tolerance factor by calculation. Perovskite precursor solutions with a concentration of about 35% in each composition are used. The solvent was a 4:1 (g/g) mix of *N*,*N*-dimethylformamide and dimethyl sulfoxide. The solution is then stirred with a magnetic stirrer for 1 h at 70 °C. The films were deposited on FTO/TiO_2_ substrates. The solution for forming substrates was made by dissolving TiO_2_ paste in ethanol, which was dropped into the FTO substrate and spread by a spin coater. The spin coating process was programmed to run at 1000 rpm for 10 s and then at 3000 rpm for another 60 s. After that, FTO/TiO_2_ substrates were thermally annealed at 100 °C for 10 min. Then, 200 μL of the precursor solution heated to 70 °C was dropped onto this substrate, and the film was deposited by using the spin coater. The spin coating process was programmed to run at 1000 rpm for 10 s and then at 2000 rpm for another 60 s. After that, the perovskite thin films were thermally annealed at 100 °C for 10 min using a hot plate.

### 2.2 Sample Characterization

To evaluate the crystallinity and lattice constants of the perovskite thin films, X-ray diffraction (XRD) patterns were acquired by using a Rigaku X-ray diffractometer. UV–vis transmission measurements were carried out by using a Shimazu SolidSpec-3700DUV spectrophotometer. Additionally, the band gap of the perovskite thin films was determined by using UV–vis spectroscopy. Absorbance was calculated from the transmittance of the light irradiated onto the samples, and the band gap was determined using Tauc plots. For the observation of the surface morphology of the perovskite thin films, a HiROX KH-7700 digital microscope was employed.

## 3 Results and Discussion

### 3.1 Calculation of Tolerance Factor and Band Gap

The tolerance factor is calculated for the sample of MA_1–*y*_FA_*y*_SnI_3–*x*_Br_*x*_ (*x* = 0–3 and *y* = 0–1 with 0.1 interval, respectively) to find the optimal composition for perovskite crystals. The values shown in Table 1 were used in the calculations.

**Table 1:** Element and Ionic Radius

element	MA^+^	FA^+^	Sn^2+^	I^–^	Br^–^
ionic radius [pm]	217	253	115^14^	206	182

To calculate the tolerance factor for samples of MA_1–*y*_FA_*y*_SnI_3–*x*_Br_*x*_, we replace the tolerance factor *t* with the following formula:^15^
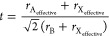
where *r*_A_effective__ and *r*_X_effective__ are the weight average ionic radii of the A and X sites, and *r*_B_ is the ionic radius of the B site. In addition, *r*_A_effective__ and *r*_X_effective__ are represented by
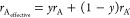

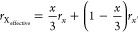


**Figure 1:**
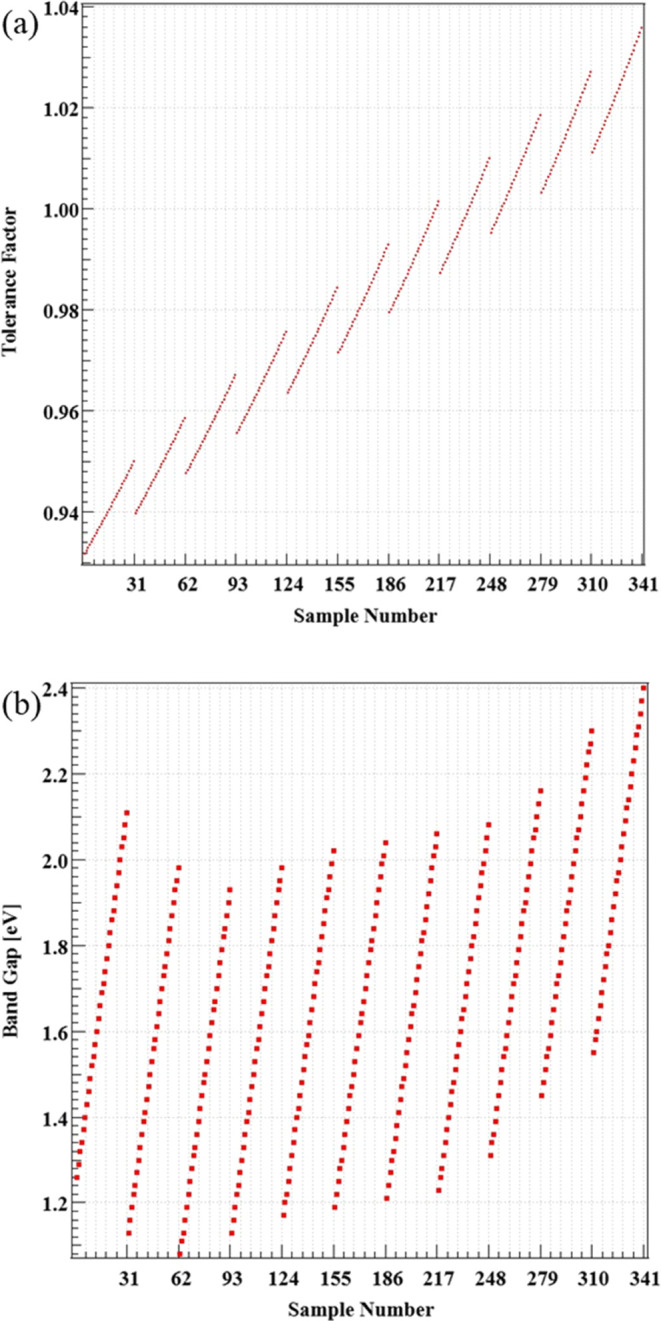
(a) Tolerance factor and (b) band gap of MA_1–*y*_FA_*y*_SnI_3–*x*_Br_*x*_ samples.

In this study, the A and X sites are occupied by two different ions; therefore, the other ions are designated as *r*_A^′^_ and *r*_*x*^′^_. Figure 1a illustrates the relationship between the tolerance factor, which was calculated using the ionic radii of each element, as shown in Table 1, and the assigned sample numbers. Table 2 illustrates the relationship between the samples and their corresponding numbers. The starting sample MASnI_3_ was assigned as sample No. 1, which was followed by samples with increasing Br. Subsequently, FA was added with an increasing content step of 0.1 each; these samples were categorized into 11 groups. Within each group, the addition of Br composition increased by 0.1 for each successive sample. During this process, adjustments were made to ensure that the first sample in each group received the next consecutive number after the last sample in the previous group. Next, we depicted the relationship between each sample and its band gap in Figure 1b. Increasing FA led to a decrease in the band gap until *x* = 0.2, after which it started increasing.^16^ Additionally, as Br was increased, the band gap linearly increased.^17^ Taking these conditions and specific values from well-studied materials like MASnI_3_ and FASnI_3_ into account, we estimated the band gap for each sample.^18−22^

**Table 2:** Sample Numbers

number	sample (*x* = 0–3 with 0.1 interval)
1–31	MASnI_3–*x*_Br_*x*_
31–62	MA_0.9_FA_0.1_SnI_3–*x*_Br_*x*_
63–93	MA_0.8_FA_0.2_SnI_3–*x*_Br_*x*_
94–124	MA_0.7_FA_0.3_SnI_3–*x*_Br_*x*_
125–155	MA_0.6_FA_0.4_SnI_3–*x*_Br_*x*_
156–186	MA_0.5_FA_0.5_SnI_3–*x*_Br_*x*_
187–217	MA_0.4_FA_0.6_SnI_3–*x*_Br_*x*_
218–248	MA_0.3_FA_0.7_SnI_3–*x*_Br_*x*_
249–279	MA_0.2_FA_0.8_SnI_3–*x*_Br_*x*_
280–310	MA_0.1_FA_0.9_SnI_3–*x*_Br_*x*_
311–341	FASnI_3–*x*_Br_*x*_

From Figure 1, it is clear that all of the calculated MA_1–*y*_FA_*y*_SnI_3–*x*_Br_*x*_ thin films have ideal tolerance factors, depending on sample compositions. Therefore, we have prepared samples of MA_0.4–0.2_FA_0.6–0.8_SnI_3–1.5_Br_0–1.5_ that are expected to have particularly ideal crystal structures and a band gap. The prepared samples were evaluated using XRD, UV–vis, and digital microscopes to obtain crystal structures and band gaps based on the sample composition.

### 3.2 Evaluation of the MA_0.4–0.2_FA_0.6–0.8_SnI_3–1.5_Br_0–1.5_ Thin Films

**Figure 2:**
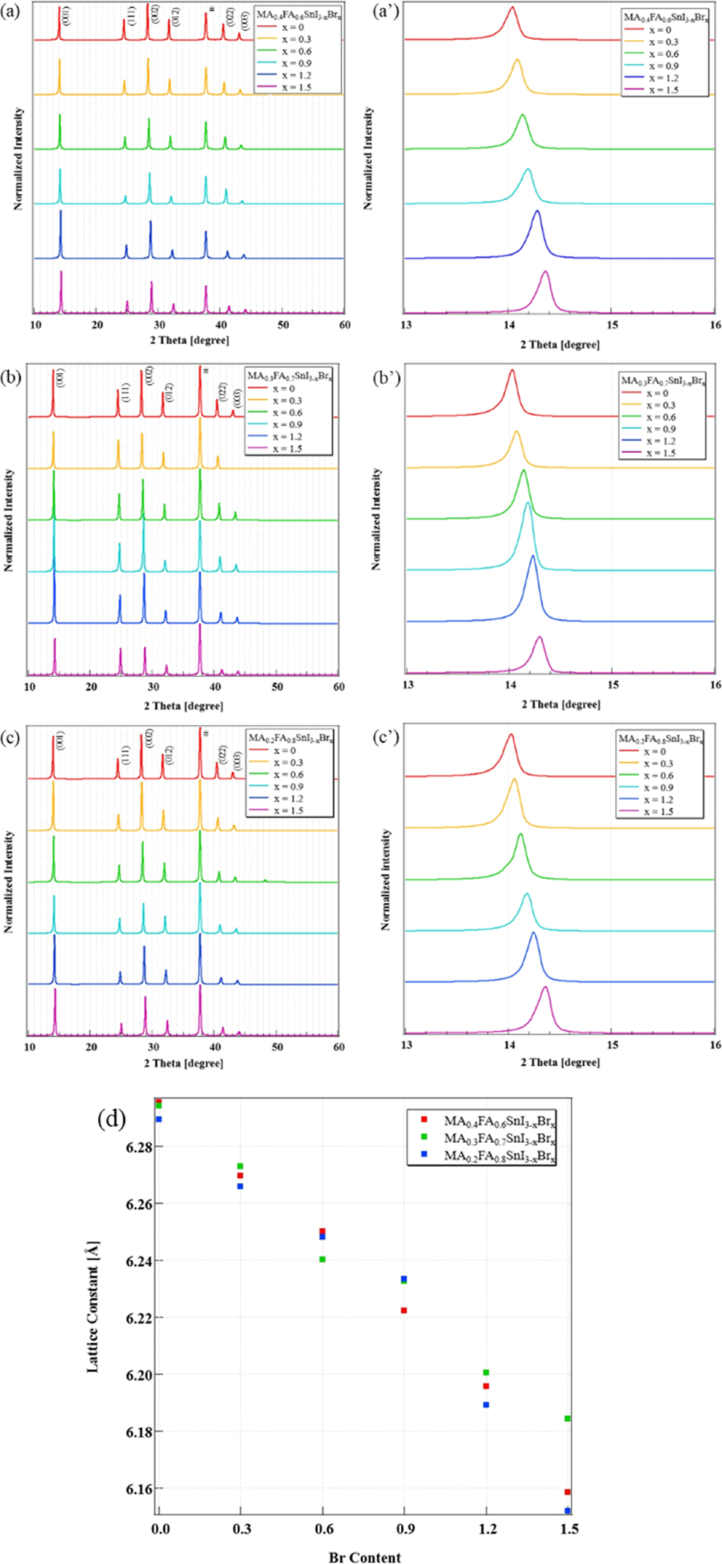
Comparison of the XRD patterns for (a, a′) MA_0.4_FA_0.6_SnI_3–1.5_Br_0–1.5_, (b, b′) MA_0.3_FA_0.7_SnI_3–1.5_Br_0–1.5_, and (c, c′) MA_0.2_FA_0.8_SnI_3–1.5_Br_0–1.5_ with (d) cubic lattice parameters.

Figure 2 shows a comparison of the X-ray diffraction patterns and lattice constants for MA_0.4–0.2_FA_0.6–0.8_SnI_3–*x*_Br_*x*_. In Figure 2a–c, the peak at 38.5° is attributed to #FTO and is utilized as a reference to normalize the perovskite diffraction peaks. It is evident from these three figures that no impurity peaks were observed in any of the samples, suggesting that the precursor solution has successfully transformed into the perovskite crystals.^23^ Furthermore, in the case of MA_0.4–0.2_FA_0.6–0.8_SnI_3–1.5_Br_0–1.5_, it was confirmed that an increase in the Br ratio led to a shift of diffraction peaks toward the higher-angle side, indicating a decrease in the lattice constant, as depicted in Figure 2a′–c′.

This phenomenon occurs due to the addition of Br, which has a smaller ionic radius than I, leading to a decrease in the lattice constants. This observation is consistent with previous studies.^17,20,22^ Additionally, as shown in Figure 2d, the lattice constant linearly decreased with the increase of Br ratio, suggesting appropriate Br supplementation in the fabricated samples.^20^ Consequently, the manufactured samples had been suitably enriched with Br, resulting in high-quality crystals. Normally, samples with a tolerance factor exceeding 1 did not exhibit a cubic crystal structure. However, as shown in Figure 2c, it was confirmed that the samples synthesized in this study, even with a calculated tolerance factor exceeding 1, still maintained a cubic crystal structure.

**Figure 3:**
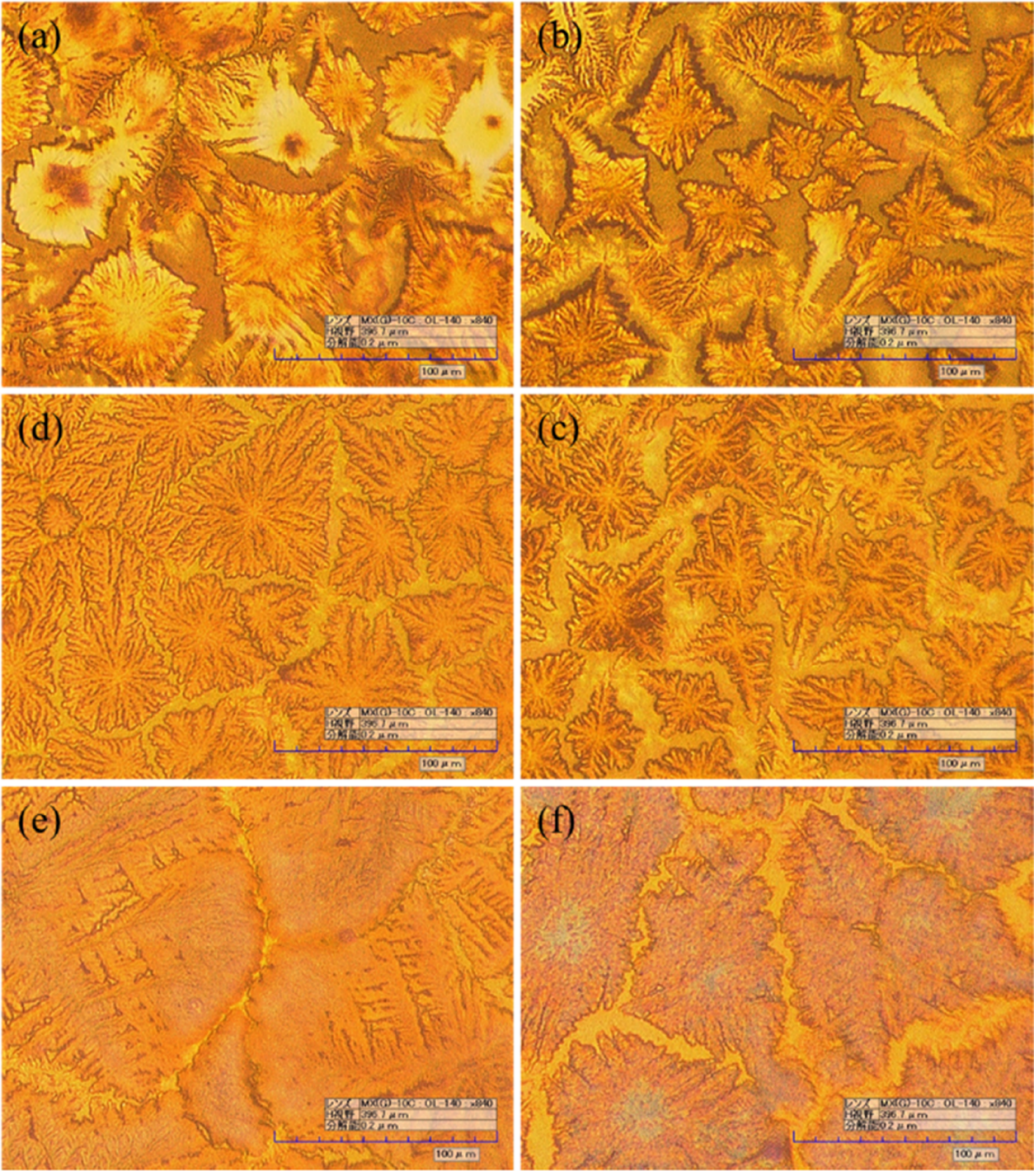
Morphology of (a) MA_0.3_FA_0.7_SnI_3_, (b) MA_0.3_FA_0.7_SnI_2.7_Br_0.3_, (c) MA_0.3_FA_0.7_SnI_2.4_Br_0.6_, (d) MA_0.3_FA_0.7_SnI_2.1_Br_0.9_, (e) MA_0.3_FA_0.7_SnI_1.8_Br_1.2_, and (f) MA_0.3_FA_0.7_SnI_1.5_Br_1.5_.

Figure 3 shows the surface morphology of the MA_0.3_FA_0.7_SnI_3–1.5_Br_0–1.5_ thin film observed by using a digital microscope. It is apparent from the figure that the substrate surface was not entirely covered by perovskite crystals, indicating the presence of voids. However, there was a trend of these voids decreasing with the increase of the Br ratio, aligning with previous research findings.^24^

A notable characteristic of Sn-based perovskite thin films is the formation of voids. This phenomenon arises from the rapid growth of perovskite crystals, resulting in the unevenness of the thin film.^25,26^ Therefore, achieving high-quality thin films should be possible by carefully controlling the addition of Br and suppressing rapid crystal growth.

On the other hand, adjusting the solvent of the precursor solution can also improve the surface morphology. Ke et al. reported that it has been observed that DMSO can slow down the crystallization speed of perovskite through the formation of the stable intermediate adduct SnI_2_·3DMSO, resulting that the films prepared from DMSO showed no pinholes.^27^ Moreover, films deposited from DMSO as the solvent were homogeneous with almost full surface coverage on the mesoporous TiO_2_ layer.^28^ Therefore, preparing a solution with an adjusted DMF/DMSO ratio would contribute to the improvement of surface coverage without increasing the amount of Br. As a result, this led to a decrease in the band gap.

**Figure 4:**
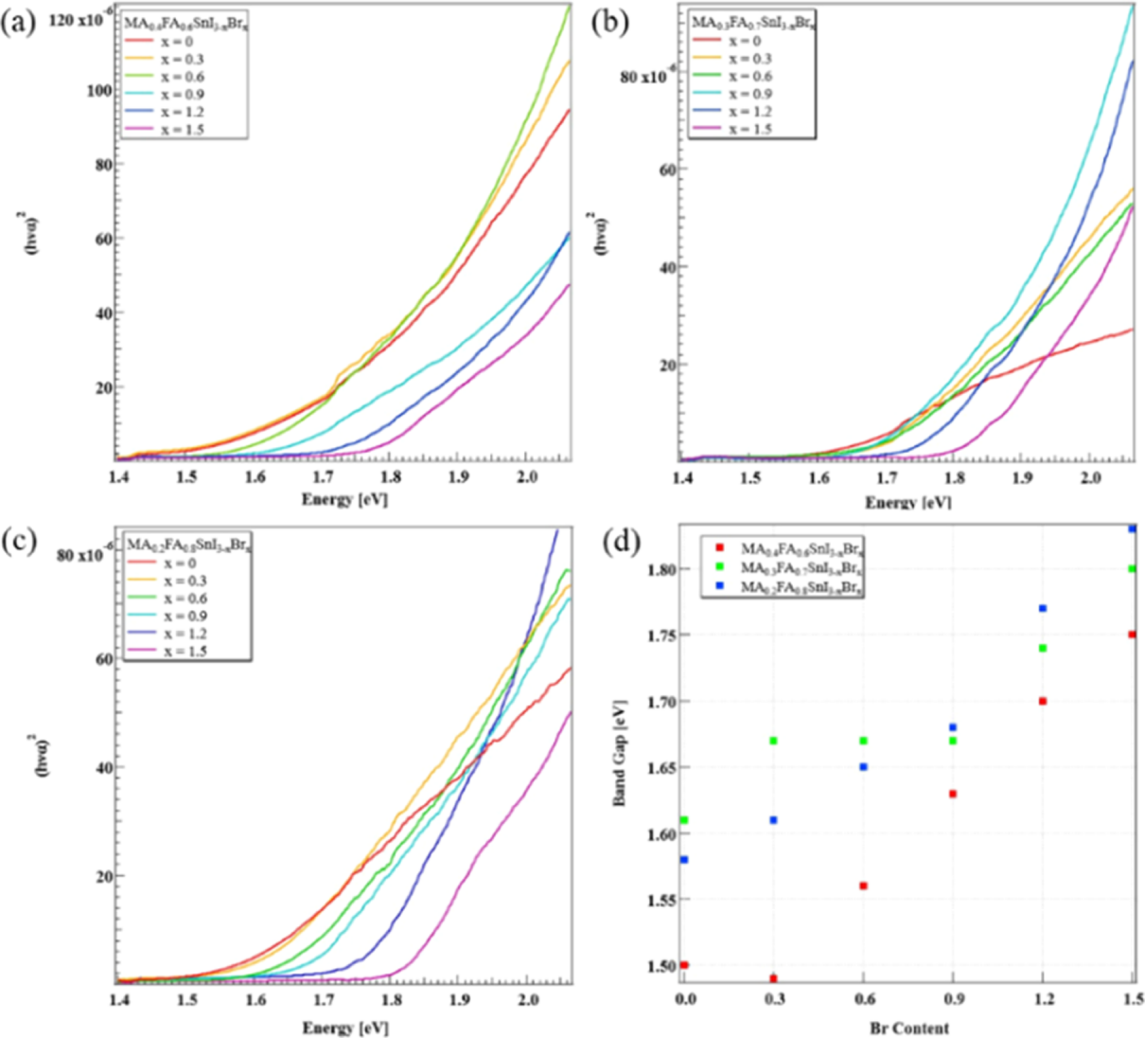
Absorption spectra of (a) MA_0.4_FA_0.6_SnI_3–1.5_Br_0–1.5_, (b) MA_0.3_FA_0.7_SnI_3–1.5_Br_0–1.5_, and (c) MA_0.2_FA_0.8_SnI_3–1.5_Br_0–1.5_. (d) Band gap for MA_0.4–0.2_FA_0.6–0.8_SnI_3–*x*_Br_*x*_ extracted from the absorption measurements depending on the content of Br.

Figure 4 shows a comparison of absorption spectra and the band gap for each sample. The band gap calculation employed Tauc plot analysis. The band gap of MA_0.4–0.2_FA_0.6–0.8_SnI_3–*x*_Br_*x*_ linearly increased with an increase in the Br ratio. However, across all samples, the experimental values were consistently approximately 0.2–0.3 eV higher than the calculated values.

One possible reason for the deviation of the band gap from the calculated value is the existence of numerous voids within the thin film. In UV–vis measurements, the absorbance is determined by detecting transmitted light. The upsurge in transmitted light results from certain areas of the substrate not being covered by perovskite crystals, leading to an increase in band gap values compared to the calculated ones. Moreover, samples with *x* = 0.9–1.5 in MA_0.3_FA_0.7_SnI_3–1.5_Br_0–1.5_ exhibited band gap values closer to the calculated ones than samples with *x* = 0–0.6. This suggests that the increased Br ratio contributes to enhanced surface coverage and reduced voids.

## 4 Conclusions

Perovskite solar cells have garnered attention due to their optimal band gap value of 1.34 eV and an ideal crystal structure closely resembling a cubic lattice. Achieving enhanced efficiency in tin-based perovskite solar cells requires the identification of an optimal composition. In this study, we calculated the optimal ratios of MA/FA and I/Br and conducted experiments to explore the optimal composition for MA_1–*y*_FA_*y*_SnI_3–*x*_Br_*x*_ thin films. While the band gap of the manufactured samples showed a trend similar to the calculated values, the experimental band gap values were higher. This difference is attributed to the characteristics of tin-based perovskite thin films, which tend to form voids. By the reduction of void formation, the thin film could achieve an appropriate band gap. Moreover, it was observed that an increase in the Br content improved the surface coverage rate but led to wider band gaps. Taking these considerations into account, the most optimal composition was concluded to be MA_0.3_FA_0.7_SnI_2.4_Br_0.6_. Additionally, adjusting the viscosity of the solution offers another approach to enhance the surface coverage rate. Therefore, it was concluded that the addition of Br, aiming for adjustments in the tolerance factor and band gap, may possibly exhibit the highest performance with a composition ratio of MA_0.2_FA_0.8_SnI_2.7_Br_0.3_. These findings indicate the possibility of developing MA_1–*y*_FA_*y*_SnI_3–*x*_Br_*x*_ thin films that are suitable for solar cell applications by carefully balancing the surface coverage rate and band gap conditions.

This article revealed the importance of the crystal growth and quality of perovskite thin films, especially for their coverage and obtaining the band gap close to the theoretical width. In addition, the calculated prospects of the band gap with different ratios of MA/FA and I/Br are relatively reliable to select the ratios of those precursor solutions to make the perovskite thin films with a focused band gap. The next step will be making the contacts on the prepared thin films to check their effect on electrical properties such as IV measurements for application to practical Pb-free perovskite solar cells.
